# Exosomes from Human Umbilical Cord Mesenchymal Stem Cells Reduce Damage from Oxidative Stress and the Epithelial-Mesenchymal Transition in Renal Epithelial Cells Exposed to Oxalate and Calcium Oxalate Monohydrate

**DOI:** 10.1155/2019/6935806

**Published:** 2019-03-19

**Authors:** Dian Li, Dan Zhang, Bo Tang, Yue Zhou, Wenhao Guo, Qing Kang, Zhang Wang, Lianju Shen, Guanghui Wei, Dawei He

**Affiliations:** ^1^Chongqing Key Laboratory of Child Urogenital Development and Tissue Engineering, Ministry of Education Key Laboratory of Child Development and Disorders, China International Science and Technology Cooperation Base of Child Development and Critical Disorders, Chongqing Key Laboratory of Pediatrics, Chongqing 400014, China; ^2^Department of Urology, Children's Hospital of Chongqing Medical University, Chongqing 400014, China

## Abstract

**Objective:**

To investigate whether exosomes from human umbilical cord mesenchymal stem cells (hUC-MSCs) can protect against the toxic effects of oxalate and calcium oxalate monohydrate (COM) crystals in human proximal tubular epithelial (HK-2) cells.

**Methods:**

Exosomes were isolated from hUC-MSCs, purified by ultracentrifugation, and verified by examination of cell morphology using transmission electron microscopy and the presence of specific biomarkers. HK-2 cells received 1 of 4 treatments: control (cells alone), hUC-MSC exosomes, oxalate+COM, or oxalate+COM and hUC-MSC exosomes. Cell viability was determined using the MTT assay. Oxidative stress was determined by measuring LDH activity and the levels of H_2_O_2_, malondialdehyde (MDA), and reactive oxygen species (ROS). Expressions of N-cadherin, TGF-*β*, and ZO-1 were determined by immunofluorescence. Expressions of epithelial markers, mesenchymal markers, and related signaling pathway proteins were determined by western blotting.

**Results:**

After 48 h, cells in the oxalate+COM group lost their adhesion, appeared long, spindle-shaped, and scattered, and the number of cells had significantly decreased. The oxalate+COM treatment also upregulated TGF-*β* and mesenchymal markers, downregulated epithelial markers, increased the levels of LDH, H_2_O_2_, MDA, and ROS, decreased cell viability, and increased cell migration. The isolated exosomes had double-layer membranes, had hollow, circular, or elliptical shapes, had diameters mostly between 30 and 100 nm, and expressed CD9, CD63, and Alix. Treatment of HK-2 cells with hUC-MSC exosomes reversed or partly reversed all the effects of oxalate+COM.

**Conclusions:**

Exosomes from hUC-MSCs alleviate the oxidative injury and the epithelial-mesenchymal transformation of HK-2 cells that is induced by oxalate+COM.

## 1. Introduction

Urinary calculi are one of the most common diseases in urology with high morbidity and recurrence rate and their formation is affected by many factors. Most kidney stones consist of calcium oxalate, and hyperoxaluria is common in patients with kidney stones. When urinary oxalate reaches a certain concentration, it can injure renal tubular epithelial cells [1, 2]. This damage often accompanies lipid peroxidation [3], which can lead to oxidative damage of epithelial cells, further promoting epithelial-mesenchymal transition, and lead to impaired renal tubular function [4]. Oxidative stress refers to a reaction state in which the excessive production of reactive oxygen species (ROS) in the body is imbalanced with the antioxidant defense mechanism under the influence of various stimulating factors [5]. It usually manifests in the production of large amounts of ROS and the reduction of some antioxidant enzymes. ROS can cause DNA breakdown by lipid peroxidation, which leads to renal damage and fibrosis [6, 7]. Patients with tubular epithelial fibrosis face a poor long-term prognosis due to the development of severe hydronephrosis, chronic kidney disease, and other conditions that lead to end-stage chronic kidney disease [8].

Mesenchymal stem cells (MSCs) are a kind of pluripotent stem cell with low immunogenicity and multidirectional differentiation ability [9]. Compared with other stem cells, they are easy to obtain and culture and have no allogeneic rejection, chemotaxis, and tissue repair. All these characteristics make them widely used in various fields such as anti-inflammatory damage and tissue damage [10] and are widely used in cell therapy [11]. Numerous studies have reported that the mechanism by which MSCs repair tissue damage is related to their paracrine function, rather than their capacity for differentiation [12, 13]. MSC exosomes, vesicles that MSCs release into the extracellular space, have roles in several intracellular signaling pathways, and they also function in the repair of cell injury [14].

MSC-derived exosomes are vesicles that can be used for the treatment of acute and chronic kidney injury [15, 16] and can also alleviate the damage from liver fibrosis [17]. This suggests that exosomes have potential as a new mode of treatment. Preliminary studies in our laboratory found that human umbilical cord MSC- (hUC-MSC-) conditioned media may help to promote the repair of renal injury [18]. However, it remains unknown whether hUC-MSC exosomes reduce oxidative stress and the EMT induced by oxalate and COM crystals (oxalate+COM) in renal tubular epithelial cells. In this study, we first demonstrated that oxalate+COM can induce the EMT in HK-2 cells and then examined the effect of hUC-MSC exosomes on protection against the numerous adverse effects of oxalate+COM.

## 2. Materials and Methods

### 2.1. Cell Culture and Treatment

The hUC-MSCs were provided by the Stem Cell Center of the Children's Hospital of Chongqing Medical University and were maintained in DMEM/F12 medium with 10% exo-free fetal bovine serum (FBS) until 80% confluence. Then, the supernatants were collected for isolation of exosomes. The exo-free fetal bovine serum was purified by ultracentrifugation, as described previously with some minor modifications [19]. Briefly, the FBS was centrifuged at 120,000 g for 10 h, and the upper 90% of the supernatant was collected as exo-free FBS.

HK-2 cells were from the Type Culture Collection of the Chinese Academy of Sciences (Shanghai) and were maintained in DMEM/F12 medium supplemented with 10% FBS and antibiotics (Gibco, USA). These cells were exposed to COM crystals (0.2 g/L) and oxalate (2 mmol/L) [20] (oxalate+COM) that were added to the culture medium for 48 h. All chemicals were from Sigma-Aldrich (USA).

To investigate the effect of hUC-MSC exosomes on HK-2 cells exposed to oxalate+COM, we examined cells that received 1 of 4 treatments: (i) control (HK-2 cells alone), (ii) hUC-MSC exosome (HK-2 cells and hUC-MSC exosomes), (iii) oxalate+COM (HK-2 cells and oxalate+COM), or (iv) oxalate+COM+hUC-MSC exosome (HK-2 cells and oxalate+COM and hUC-MSC exosomes). The control group and oxalate+COM group were treated as described above. Based on our initial experiments, 160 *μ*g/mL hUC-MSC exosomes were used for the hUC-MSC exosome group and the oxalate+COM+hUC-MSC exosome group.

### 2.2. Isolation of Exosomes

HUC-MSC exosomes were isolated by differential centrifugation, as previously described with some minor modifications [16]. Briefly, exo-free medium was centrifuged at 300 g for 30 min, 3000 g for 30 min, and then 6000 g for 30 min for removal of cell debris. The supernatant was loaded onto a 30% sucrose/D_2_O cushion and then ultracentrifuged at 120,000 g for 90 min. The D_2_O cushion was collected and washed 2 times with phosphate-buffered saline (PBS) by centrifugation at 120,000 g for 90 min. All centrifugation procedures were performed at 4°C. The purified exosomes were resuspended in DMEM/F12 medium and passed through a 0.22 mm filter prior to use in experiments.

### 2.3. Transmission Electron Microscopy

Transmission electron microscopy (TEM) was used to examine the morphology of the exosomes. A drop of exosomes (20 *μ*L) was added to a grid that was coated with formvar and carbon and incubated for 30 min at room temperature. All excess fluid was removed using a filter paper, and the samples were negatively stained with uranyl acetate for 30 s. The samples were then air-dried using an electric incandescent lamp and viewed using an electron microscope (Hitachi, S-3000N).

### 2.4. Nanoparticle Tracking Analysis

All samples were diluted in PBS so the particle concentration was suitable for analysis by nanoparticle tracking analysis (NTA), which was performed using the Zetasizer Nano ZS90 (Malvern Panalytical, England). Then, about 1000 mL was injected into the LM10 unit (Malvern Panalytical). Videos were collected and analyzed using NTA software, version 2.3 (Malvern Panalytical, England).

### 2.5. Exosome Labeling and Tracking in HK-2 Cells

HUC-MSC exosomes were labeled with the membrane dye, PHK26 (Sigma-Aldrich, USA), according to the manufacturer's protocol. HK-2 cells were seeded in 24-well plates (Corning Incorporated, USA) at 3 × 10^4^ cells per well. Then, 160 *μ*g/mL PKH26-labeled hUC-MSC exosomes were added for 6, 12, 24, or 48 h at 37°C. The cells were then washed with PBS and fixed in 4% paraformaldehyde for 20 min. The nuclei were counterstained with DAPI (1 : 200). Cells were visualized using a fluorescence microscope (Nikon, Japan).

### 2.6. Western Blot Analysis

Protein samples of cells and exosomes were lysed in RIPA lysis buffer (Beyotime, China) with phenylmethanesulfonyl fluoride (PMSF; Beyotime, China) and centrifuged at 12,000 g for 20 min at 4°C. The protein concentration was determined using a bicinchoninic acid (BCA) assay kit (Beyotime, China). Then, 50 *μ*g of total lysate was diluted in the sodium dodecyl sulfate polyacrylamide gel electrophoresis (SDS-PAGE) sample buffer, loaded onto SDS-PAGE gels, subjected to electrophoresis, and then transferred to polyvinylidene difluoride (PVDF) membranes (Millipore, USA). Membranes were blocked in 5% nonfat milk for 1 h and then incubated with different monoclonal primary antibodies overnight (rabbit anti-CD63 [1 : 2000, Abcam, USA], rabbit anti-CD9 [1 : 500, Abcam, USA], mouse anti-Alix [1 : 1000, Abcam, USA], mouse anti-vimentin [1 : 1000, Bioster, China], mouse anti-N-cadherin [1 : 1000, Invitrogen, USA], mouse anti-TGF-*β* [1 : 1000, Abcam], mouse anti-ZO-1[1 : 1000, Invitrogen, USA], mouse anti-E-cadherin [1 : 1000, Abcam, USA], mouse anti-Smad2 [1 : 1000, Abcam, USA], and mouse anti-*β*-actin [1 : 1000, Cell Signaling, USA] as a loading control). After washing in tris-buffered saline/Tween (TBST), the membranes were incubated with goat anti-rabbit or mouse antibodies (1 : 1000, Zhongshan, China) for 2 h at 37°C. The immunoblots were visualized using the Immobilon Western Chemiluminescent HRP Substrate (Millipore, USA), and the bands were quantified by densitometric analysis (GeneGnome, USA), relative to *β*-actin.

### 2.7. MTT Assay

We used the 3-(4,5-dimethylthiazol-2-yl)-2,5-diphenyltetrazolium bromide (MTT; Solarbio, China) assay to determine the proliferative potential of HK-2 cells, as described previously [16, 21]. Briefly, HK-2 cells were seeded in 96-well plates, with 5 × 10^3^ cells and 100 *μ*L of medium with 10% FBS per well. The four groups (described above) were incubated for 24, 48, or 72 h. The culture medium was removed, and 20 *μ*L of 5 mg/mL MTT reagent and 80 *μ*L of serum-free medium were added to each well. After incubation at 37°C for 4 h, the supernatant was removed, and 150 *μ*L of dimethylsulfoxide (DMSO; Solarbio, China) was added to each well. After shaking for 5 min, the absorbance was measured at 492 nm.

### 2.8. Oxidative Stress Assays

We measured the enzymatic activity of lactate dehydrogenase (LDH; Solarbio, China) and the concentrations of malondialdehyde (MDA; Beyotime, China) and H_2_O_2_ using appropriate colorimetric assay kits (Elabscience, China).

### 2.9. Measurement of Intracellular Reactive Oxygen Species

The level of intracellular reactive oxygen species (ROS) was measured using the 2′,7′-dichlorofluorescein diacetate (DCFH-DA) assay (Sigma-Aldrich, USA), in which the intensity of fluorescence is proportional to ROS level. HK-2 cells were first seeded on 6-well plates. When they reached 70 to 80% confluence, they were treated with the relevant medium for 48 h. Then, cells were washed twice with PBS and DCFH-DA was added. Fluorescence was measured using a fluorescence microscope, after incubation for 20 min in the dark at 37°C.

### 2.10. Immunofluorescence Staining

HK-2 cells were seeded on cell slides in 24-well plates at 5 × 10^4^ cells per well. Cells were then washed with PBS, fixed with 4% paraformaldehyde for 20 min, and then permeabilized and blocked with 0.3% Triton X-100 in 0.5% BSA for 1 h. The cells were then incubated overnight at 4°C with primary antibodies (1 : 50) against N-cadherin and ZO-1 (Invitrogen, USA). The Cy3 or Alexa Fluor® 488-labeled secondary IgG (1 : 200) was added for 1 h, and the cells were then incubated with DAPI (Beyotime, China) for 45 min. The cell slides were examined under a fluorescence microscope (Nikon, K10587, Japan).

### 2.11. Wound Healing Assay

The cells were seeded in 6-well plates at 3 × 10^5^ cells per well. When they reached 70 to 80% confluence, the cell monolayer was then “wounded” using a 200 *μ*L pipette tip and then washed twice with PBS. The cells were then treated as previously described [21]. Photographs were taken after 48 h. Initial and final wound widths were measured using NIS-Elements (Nikon, Japan) measurement analysis software, and the difference was determined using the following formula: (initial wound width - final wound width)/2.

### 2.12. Statistical Analysis

All data are expressed as means ± standard deviations (SDs). All statistical analysis was performed using GraphPad Prism, version 6.0. The means of different treatment groups were compared using a one-way analysis of variance (ANOVA). A result was considered significant if the *p* was below 0.05.

## 3. Results

### 3.1. Oxalate and COM Crystals Induce the EMT in HK-2 Cells

**Figure 1:**
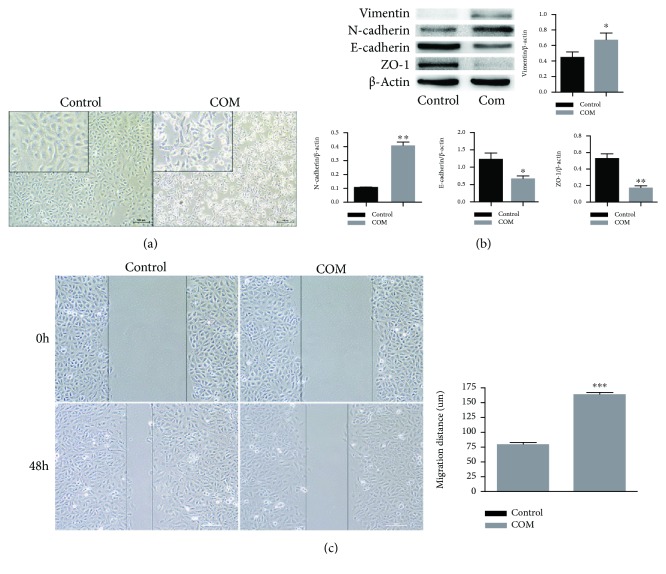
Induction of the EMT in HK-2 cells by incubation with oxalate+COM. (a) Morphology of HK-2 cells incubated for 48 h without (control) or with oxalate+COM. (b) Western blotting of ZO-1, E-cadherin, vimentin, and N-cadherin in HK-2 cells incubated for 48 h without or with oxalate+COM. (c) Migration of HK-2 cells incubated for 48 h without or with oxalate+COM. These results were representative of at least three independent experiments. Results were considered statistically significant *P* < 0.05 (^∗^*P* < 0.05, ^∗∗^*P* < 0.01, and ^∗∗∗^*P* < 0.001 vs. the control group).

**Figure 2:**
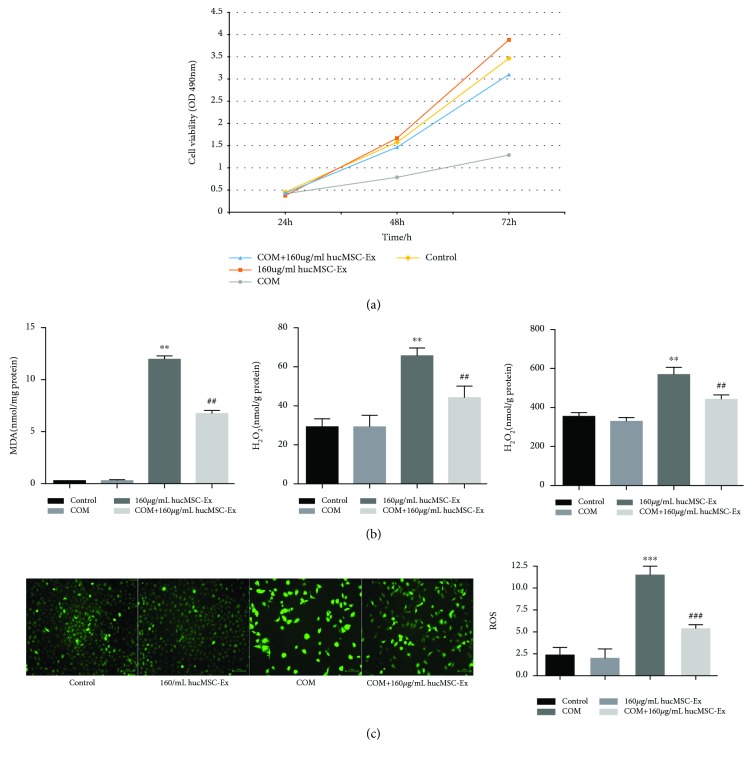
hUC-MSC exosomes restore the viability and prevent oxidative injury of HK-2 cells. (a) Cell viability in each treatment group at 24, 48, and 72 h. (b) Levels of LDH, MDA, and H_2_O_2_ at 48 h in each treatment group. (c) ROS levels (DCFH-DA staining) at 48 h in each treatment group. These results were representative of at least three independent experiments. Results were considered statistically significant *P* < 0.05 (^∗∗^*P* < 0.01 and ^∗∗∗^*P* < 0.001 vs. the control group; ^##^*P* < 0.01 and ^###^*P* < 0.001 vs. the oxalate+COM group).

We first confirmed that oxalate and COM crystals can induce the EMT in HK-2 cells. Thus, we cocultured the cells without (control) or with oxalate+COM for 48 h and then examined cell morphology, epithelial and mesenchymal markers, and cell migration (Figure 1). The results show that the control (untreated) cells were small and round and had a paving stone-like structure (Figure 1(a)), with obvious expression of ZO-1 at the cell junctions (Figure 1(b)). However, the oxalate+COM treatment decreased cell viability (Figure 2(a)), increased the intercellular spaces, and reduced cell adhesion; these cells were also long and spindle-shaped and had irregular morphology (Figure 1(a)). The oxalate+COM treatment also increased the expression of vimentin and N-cadherin, but decreased the expression of E-cadherin and ZO-1 (Figure 1(b)). The cell migration assay demonstrated that the oxalate+COM treatment significantly promoted cell migration (Figure 1(c)). These findings indicate that the oxalate+COM treatment promotes the EMT of HK-2 cells, consistent with previous reports.

### 3.2. HK-2 Cells Internalize hUC-MSC Exosomes

**Figure 3:**
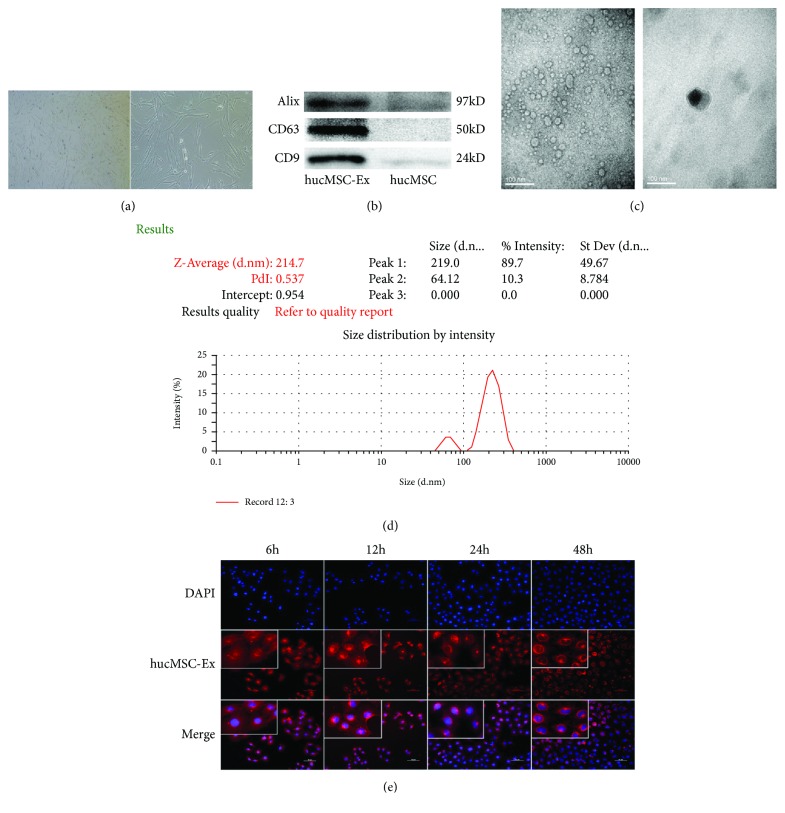
Characterization and internalization of hUC-MSC exosomes. (a) Morphology of hUC-MSCs in exo-free medium. (b) Western blotting for exosome markers in hUC-MSC exosomes and hUC-MSCs. (c) Morphology of hUC-MSC exosomes under transmission electron microscopy. (d) NTA of hUC-MSC exosomes. (e) Fluorescence microscopy of HK-2 cells and hUC-MSC exosomes after coincubation for 6, 12, 24, and 48 h (left to right). These results were representative of at least three independent experiments.

We successfully purified hUC-MSC exosomes by differential centrifugation on a 30% sucrose/D_2_O cushion and then diluted the exosome suspension by 20-fold in PBS and observed them using TEM. The exosomes had the typical circular, oval, or saucer-like shape, with obvious heterogeneity; most had diameters of 30 to 100 nm and they contained low-density material (Figure 3(c)). Examination of exosomal markers indicated an abundance of Alix, CD63, and CD9 (Figure 3(b)). The NTA showed that the diameters of the exosomes ranged from 80 to 300 nm, with most of them between 100 nm and 300 nm (Figure 3(d)).

We also examined whether HK-2 cells can internalize hUC-MSC exosomes. Thus, we added 80 *μ*g/mL of PKH-26-labeled exosomes (which emit red fluorescence) into the culture medium and incubated them with HK-2 cells in 24-well plates for 6, 12, 24, or 48 h at 37°C (Figure 3(e)). The results show internalization of the exosomes into the cytoplasm of HK-2 cells after 6 and 12 h. At 24 h, the exosomes started to aggregate along the nuclear membrane and they remained around the nucleus at 48 h.

### 3.3. hUC-MSC Exosomes Restore Viability and Reduce Oxidative Injury of HK-2 Cells

We used the MTT assay to measure the viability of HK-2 cells in each group at 24, 48, and 72 h (Figure 2(a)). At 24 h, the 4 groups had no significant differences in cell viability. However, at 48 h and 72 h, the viabilities of the control group, hUC-MSC exosome group, and oxalate+COM+hUC-MSC exosome group were all higher than those of the oxalate+COM group (*P* < 0.01 for all comparisons). This result indicates that the oxalate+COM treatment decreased viability and that hUC-MSC exosomes reversed this effect (Figure 2(a)). In addition, measurement of intercellular MDA, H_2_O_2_, and LDH activity in HK-2 cells at 48 h indicated that the oxalate+COM treatment increased oxidative stress and that hUC-MSC exosomes reversed this effect (*P* < 0.01) (Figure 2(b)). In agreement, measurement of ROS by DCFH-DA (green fluorescent dye) at 48 h indicated increased fluorescence following the oxalate+COM treatment and reversal of this effect by addition of hUC-MSC exosomes (Figure 2(c)).

### 3.4. hUC-MSC Exosomes Inhibit the Oxalate+COM-Induced EMT of HK-2 Cells

**Figure 4:**
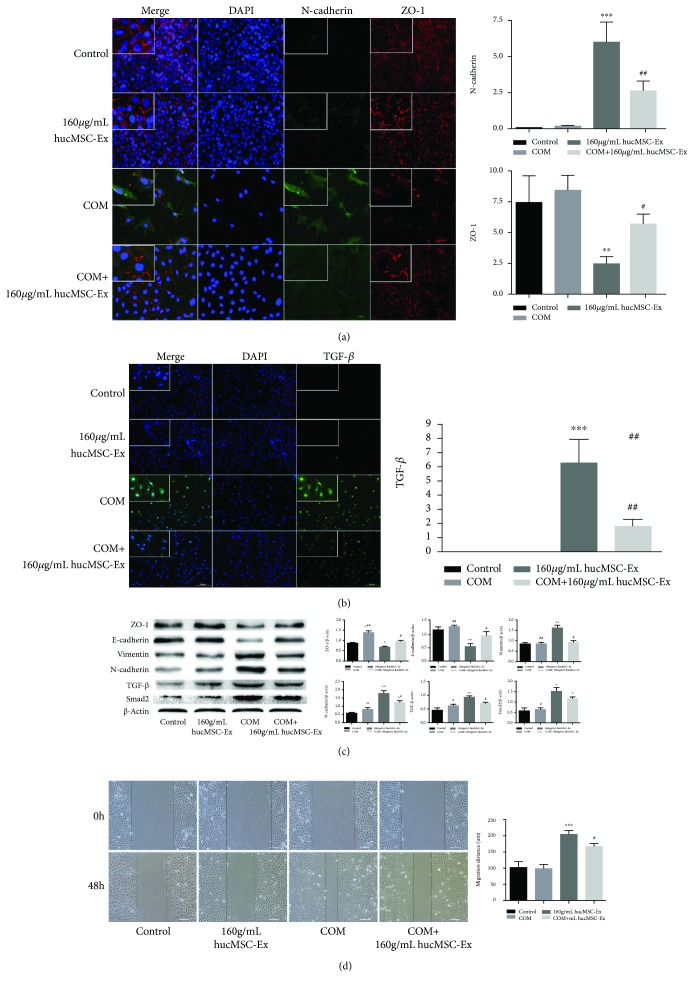
hUC-MSC exosomes inhibit oxalate+COM-induced EMT of HK-2 cells. (a) Expression of ZO-1 and N-cadherin at 48 h in each treatment group. (b) Expression of TGF-*β* by HK-2 cells at 48 h in each treatment group. (c) Western blotting of ZO-1, E-cadherin, vimentin, N-cadherin, TGF-*β*, and Smad2 at 48 h in HK-2 cells of each treatment group. (d) Cell migration of each treatment group at 48 h. These results were representative of at least three independent experiments. Results were considered statistically significant *P* < 0.05 (^∗^*P* < 0.05, ^∗∗^*P* < 0.01, and ^∗∗∗^*P* < 0.001 vs. the control group; ^#^*P* < 0.05 and ^##^*P* < 0.01 vs. the oxalate+COM group).

We also studied the effect of hUC-MSC exosomes on the EMT of HK-2 cells that were stimulated by oxalate+COM. Thus, we measured the expression of ZO-1 and N-cadherin by immunofluorescence in each group after 48 h (Figure 4(a)). The results indicate that the oxalate+COM treatment increased the level of cytoplasmic and nuclear N-cadherin, but decreased the level of cytoplasmic ZO-1. However, addition of hUC-MSC exosomes reversed these effects. Immunofluorescence of TGF-*β* indicated a pattern similar to that of N-cadherin (Figure 4(b)).

We then measured the expression of E-cadherin, ZO-1, vimentin, N-cadherin, and related signaling proteins (TGF-*β* and Smad2) by western blotting (Figure 4(c)). The results indicate decreased levels of mesenchymal markers (vimentin, N-cadherin) and increased levels of epithelial markers (E-cadherin, ZO-1) in oxalate+COM cells relative to the controls and that hUC-MSC exosomes reversed this effect. The same pattern occurred in the signaling pathway proteins TGF-*β* and Smad2.

In agreement, cell migration assays indicated that control cells did not migrate to the adjacent region of the assay device, addition of oxalate+COM-induced cell migration to the adjacent region, and that hUC-MSC exosomes reversed the effect of oxalate+COM (Figure 4(d)).

## 4. Discussion

Kidney stones are composed of various materials, and calcium oxalate is the most common component. Kidney stones are often accompanied by lipid peroxidation due to the injury of HK-2 cells by oxalate+COM [1]. Previous research found that H_2_O_2_-induced chronic oxidative injury and accumulation of advanced oxidation protein products (AOPPs) can cause the EMT in HK-2 cells [22]. A previous study [23] reported that oxalate+COM can directly induce the EMT in HK-2 cells. In the present study, we found that incubation of HK-2 cells with oxalate+COM decreased the expression of epithelial markers and increased the expression of mesenchymal markers. This phenomenon is consistent with these previous studies and indicates our successful establishment of this *in vitro* model of the EMT in HK-2 cells.

Exosomes have an important role in intercellular communications and may be involved in the repair process that stem cells promote [13, 24]. Therefore, we examined whether hUC-MSC exosomes can inhibit the EMT in HK-2 cells. First, we isolated hUC-MSC exosomes by differential centrifugation. The structure, markers, and NTA analysis of these exosomes were consistent with previous studies [14]. Our successful isolation of these exosomes allowed us to examine their effects on HK-2 cells.

Lipid peroxidase (LPO) metabolizes polyunsaturated fatty acids to MDA [25], and the reaction of ROS, such as H_2_O_2_, with polyunsaturated fatty acids also leads to the production of MDA. This damage of the side chain of polyunsaturated fatty acids within cell membranes alters membrane fluidity and permeability. This eventually leads to changes in cell structure and function and the overproduction of intracellular LDH.

High concentrations of oxalate+COM cause ROS injury in renal epithelial cells and this is important in the pathogenesis of kidney stone formation and tissue damage. Therefore, removal of ROS by antioxidants may help to protect the kidney. In fact, removal of ROS is a novel idea for the prevention and treatment of kidney stones [26, 27]. We found that oxalate+COM induces ROS injury in HK-2 cells and that hUC-MSC exosomes partially reversed this effect, by increasing cell viability and reducing the levels of MDA, H_2_O_2_, and LDH. Thus, MSC exosomes can effectively reduce the ROS-induced injury of HK-2 cells *in vitro* and help to maintain their integrity.

The EMT plays a crucial role in renal fibrosis. In this process, epithelial cells transform into myofibroblasts following tissue injury and these cells lose their tight junctions, intercellular adhesion, and apicobasal polarity [28]. The cells then acquire the characteristics of mesenchymal cells, with downregulation of the epithelial marker E-cadherin and the tight junction protein ZO-1, leading to reduced cell contact and division. At the same time, the EMT leads to upregulation of mesenchymal markers, such as vimentin and N-cadherin [29].

Our results indicated that coculture of hUC-MSC exosomes with HK-2 cells that were induced by oxalate+COM significantly increased the expression of E-cadherin and ZO-1, but their expression remained lower than that in the control cells. These oxalate+COM-induced HK-2 cells overexpressed N-cadherin and vimentin, indicating they had already begun the EMT. However, the exosome treatment significantly ameliorated the effect of oxalate+COM.

At the same time, we found that the classical EMT-inducible signaling molecules—TGF-*β*/Smad2—were upregulated in HK-2 cells induced by oxalate+COM. Thus, oxalate+COM appears to activate this pathway, and exosomes partly ameliorate this response. TGF-*β* and the downstream Smad2 molecules play an important role in the EMT process [30]. Our previous study indicated that hUC-MSC-conditioned medium attenuates interstitial fibrosis and stimulates the repair of tubular epithelial cells by reducing the expression of TGF-*β* in kidney tissue [18]. Exosomes may be responsible for this protective effect, and the present study was an attempt to verify this hypothesis. In addition, one study reported that human urine-derived stem cell exosomes express high levels of BMP-7 [31], and another study [23] found that these stem cells can promote the EMT of HK-2 cells incubated with oxalate+COM by inhibiting the expression of BMP-7. BMP-7 is in the TGF-*β* superfamily and can antagonize TGF-*β* [32], reduce cell apoptosis, and inhibit the EMT. Therefore, we speculate that hUC-MSC exosomes protect cells by producing BMP-7. However, we have not yet examined the specific mechanism by which hUC-MSC exosomes protect against cell injury. Thus, our results must first be confirmed by animal experiments before we can consider it as a clinical treatment for kidney stones and chronic kidney disease.

## 5. Conclusions

In summary, the present *in vitro* study indicated that hUC-MSC exosomes can alleviate the oxalate+COM-induced ROS injury and EMT in HK-2 cells. This effect may be due to an exosome antagonist of TGF-*β*, although further studies are needed to identify the specific mechanism.

## Data Availability

The data used to support the findings of this study are available from the corresponding author upon request.
